# Navigating first- and second-line treatment options in hormone receptor-positive HER2-negative advanced breast cancer

**DOI:** 10.1093/oncolo/oyag215

**Published:** 2026-06-09

**Authors:** Jose Sandoval, Patrick Neven, Anna Emde, François-Clément Bidard, Philippe Aftimos, Antonio Llombart, Eva Ciruelos, Peter A Fasching, Huiping Li, Mario Campone, Monique Coersmeyer, Stefanie Srock, Michael Gnant, Carlos Barrios

**Affiliations:** Faculty of Medicine, Geneva University and Department of Medical Oncology, Geneva University Hospitals, Geneva, 1205, Switzerland; Department of Oncology, Gynecologic Oncology and Multidisciplinary Breast Center, University Hospitals Leuven, Leuven, 3000, Belgium; International Business Unit Oncology, Eli Lilly Israel, Ra’anana, 4366411, Israel; Institut Curie, UVSQ and Paris Saclay University, Saint Cloud, 92210, France; Institut Jules Bordet, Université Libre de Bruxelles, Brussels, 1070, Belgium; Valencia Hospital Arnau Vilanova and Universidad CEU Cardenal Herrera, Moncada, 46015, Spain; Medical Oncology Department, Breast Cancer Unit, Hospital Universitario 12 de Octubre, Madrid, 28041, Spain; University Hospital Erlangen Comprehensive Cancer Center Erlangen-EMN, Friedrich-Alexander University Erlangen-Nuremberg, Erlangen, 91054, Germany; Key Laboratory of Carcinogenesis and Translational Research (Ministry of Education), Department of Breast Oncology, Peking University Cancer Hospital & Institute, Beijing, 100142, China; Institut de Cancérologie de l'Ouest (ICO), Angers/Saint Herblain, 49055/44805, France; CRCI^2^NA – Inserm U1307, CNRS UMR6075, Nantes Université, Université d'Angers, Nantes, 44805, France; Global Medical Affairs, Eli Lilly (Suisse) S.A., Vernier, 1214, Switzerland; Medical Affairs Germany, Austria, Switzerland, Lilly Deutschland GmbH, Bad Homburg, D-61352, Germany; Comprehensive Cancer Center, Medical University of Vienna, Vienna, 1090, Austria; Austrian Breast and Colorectal Cancer Study Group, Vienna, 1190, Austria; Latin American Cooperative Oncology Group (LACOG), Porto Alegre, 90619-900, Brazil

**Keywords:** hormone receptor-positive, advanced breast cancer, sequencing, oral SERD, antibody-drug conjugate

## Abstract

Outcomes for patients with hormone receptor-positive HER2-negative (HR+ HER2–) advanced breast cancer (ABC) have improved considerably in recent years and CDK4/6 inhibitors are the preferred first-line therapy for most patients. Newer therapeutic classes include PI3K/AKT inhibitors, oral selective estrogen receptor degraders, proteolysis-targeting chimeras, PARP inhibitors, and antibody-drug conjugates. The development and regulatory approval of these new therapies to treat HR+ HER2– breast cancer raises the question of how to use and sequence this fast-growing armamentarium to maximize benefit for individual patients. Disease progression and resistance mechanisms emerging on treatment (therapeutic pressure) add further complexity by inducing molecular alterations; thus, a clear understanding of the mechanisms behind resistance to both endocrine and CDK4/6 inhibitor therapies and their implications for subsequent treatment is critical. In principle, the most effective and tolerable drugs should be used first with the goals of delaying chemotherapy and offering patients the best quality and duration of life. This review aims to explain the evolving treatment landscape for HR+ HER2– ABC and provides the scientific background for developing future treatment algorithms driven by preclinical and clinical results.

Implications for Practice The plethora of new agents to treat hormone receptor-positive HER2-negative breast cancer provides an array of treatment strategies for clinicians, raising the question of how to use and sequence this fast-growing armamentarium to maximize benefit for individual patients. Molecular changes associated with disease progression and emerging resistance add further complexity and are important to consider when selecting treatment strategies. This review aims to explain the evolving treatment landscape and provides the scientific background for developing future treatment algorithms driven by preclinical and clinical results.

## Introduction

In recent years, treatment of advanced breast cancer (ABC) has become increasingly personalized. Targets and critical pathways beyond hormone receptor (HR) and HER2 status have been identified, expanding treatment options and our understanding of endocrine sensitivity and resistance. Nevertheless, the estrogen receptor pathway remains the primary driver of luminal disease. In most patients with HR-positive disease, international guidelines^1–3^ recommend exhausting all available estrogen receptor-targeting options before switching to cytotoxic chemotherapy or antibody-drug conjugates (ADCs), as they combine substantial treatment benefits with a lower treatment burden on patients and reduced impact on quality of life.

**Figure 1.**
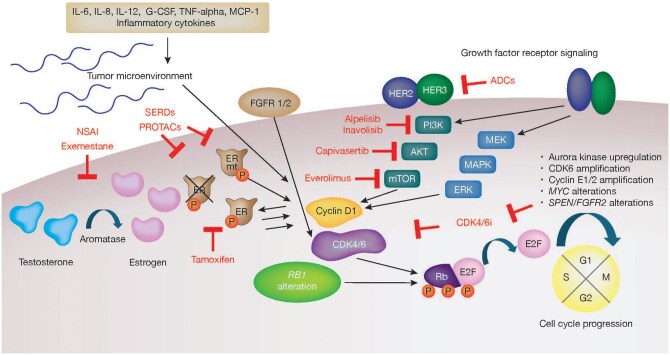
Mechanisms of resistance and impact on efficacy across different drug classes. Abbreviations: ADCs, antibody-drug conjugates; CDK4/6i, CDK4/6 inhibitor; ER, estrogen receptor; ERK, extracellular signal-regulated kinase; ER mt, estrogen receptor-mutant; FGFR, fibroblast growth factor receptor; G-CSF, granulocyte colony-stimulating factor; IL, interleukin; MAPK, mitogen-activated protein kinase; MCP-1, monocyte chemoattractant protein-1; MEK, mitogen-activated protein kinase kinase; mTOR, mammalian target of rapamycin; NSAI, non-steroidal aromatase inhibitor; PI3K, phosphatidylinositol-3 kinase; PROTAC, proteolysis-targeting chimera; SERD, selective estrogen receptor degrader; TNF, tumor necrosis factor.

In this article, an invited group of breast cancer experts reviews key trials leading to global guideline recommendations to help oncologists navigate treatment options for endocrine-resistant HR-positive HER2-negative (HR+ HER2–) ABC, considering known mechanisms of endocrine therapy (ET) resistance (Figure 1)^4–10^ and the potential of molecular testing to refine treatment decision-making. A CDK4/6 inhibitor (CDK4/6i)-containing regimen can be considered standard of care for most CDK4/6i-naïve patients with HR+ HER2– ABC (Table S1).^2^^,^^6^^,^^11–32^ CDK4/6is are used broadly in HR+ HER2– disease, improving outcomes irrespective of *ESR1* or *PIK3CA* mutation status.^19^^,^^29–32^  *ESR1* mutations play an important role in acquired resistance to aromatase inhibitors (AIs),^32^^,^^33^ but mechanisms of de novo and acquired resistance to CDK4/6i have not been fully elucidated.^34^

Following progression on a CDK4/6i and ET, optimal treatment sequencing remains undefined. Understanding mechanisms of resistance is essential if novel therapeutic strategies are to be developed and utilized optimally.^34–36^ In real-world analyses, at least one genomic alteration (*ESR1*, *PIK3CA*, *AKT1*, or *PTEN* mutation/loss) was detected in approximately 60% of HR+ HER2– tumors in the first-line metastatic setting, with increasing *ESR1* mutations through subsequent treatment lines.^37^ Knowledge of the tumor mutational status is important for treatment selection,^38^ while also considering patient comorbidities and preferences when sequencing endocrine-based therapies.^3^

## Anti-endocrine treatments

### ET + CDK4/6i

For some patients, re-exposure to a CDK4/6i and ET by switching one component may be reasonable. Statistically significant but clinically modest progression-free survival (PFS) benefits were demonstrated with continuation of a CDK4/6i (and switched ET) in both the MAINTAIN randomized phase 2 trial (ribociclib)^39^ and the postMONARCH randomized phase 3 trial (abemaciclib).^40^ In postMONARCH, PFS benefit was observed regardless of the duration of prior CDK4/6i therapy or *ESR1* or *PIK3CA* mutation status,^40^ and appeared to be greater in patients without visceral metastases. Real-world data suggested similar outcomes with second-line abemaciclib regardless of the type of first-line CDK4/6i, supporting abemaciclib retreatment in abemaciclib-pretreated ABC.^41^ In contrast, PFS was not improved by continuing palbociclib while switching ET versus switching ET alone,^42^ or by adding palbociclib to fulvestrant following progression on a CDK4/6i.^43^ Exploratory genomic analyses of real-world data hint that *TP53* mutations, *CDK4* amplifications, and *RB1* or *FAT1* loss-of-function mutations may predict failure of CDK4/6i retreatment.^44^ CDK4/6i safety profiles are characterized by neutropenia and interstitial lung disease/pneumonitis,^45–47^ together with diarrhea, hepatotoxicity, and venous thromboembolism with abemaciclib,^46^ and severe cutaneous adverse reactions, QT interval prolongation, and hepatotoxicity with ribociclib.^47^

### ET + everolimus

Another approach is to combine ET with the mTOR inhibitor everolimus. In the BOLERO-2 trial, PFS (but not overall survival [OS]) was significantly improved by adding everolimus to exemestane in AI-resistant ABC, regardless of alterations in *PIK3CA*, *FGFR1*, and *CCND1* or their pathways (Table 1).^48–51^ In the evERA control arm, everolimus plus ET demonstrated median PFS of 5.5 months in CDK4/6i-pretreated patients, irrespective of *ESR1* mutation status.^62^ Consistent data were seen with everolimus plus ET in the ESME-MBC real-world dataset (median time to progression of 5.2 months).^63^ Specific adverse effects of everolimus include noninfectious pneumonitis, infections, severe hypersensitivity reactions, angioedema (particularly in patients taking concomitant angiotensin converting-enzyme inhibitors), stomatitis, renal failure, impaired wound healing, metabolic disorders (requiring serum glucose and lipid monitoring), and myelosuppression.^64^

**Table 1.** Summary of key clinical trials evaluating PI3K/AKT pathway inhibitors.

Study name	Setting	Biomarker	Eligibility	Treatment	Endpoints	Hazard ratio (95% CI)	Translational findings
**BOLERO-2** ^48^ ^,^ ^49^	1L+	All comers	Postmenopausal, AI refractory, ≤1 prior line of CT	Everolimus + exemestane (*n = *485) Placebo + exemestane (*n = *239)	Primary: PFS Secondary: OS	PFS: 0.43 (0.35-0.54; *P <* .001) OS: 0.89 (0.73-1.10; *P =* .14)	Benefit regardless of alterations in *PIK3CA*, *FGFR1*, and *CCND1* or their pathways^50^^,^^51^
**SOLAR-1** ^52^ ^,^ ^53^	1-2L	*PIK3CA*m for primary endpoint	Prior AI, no prior CT for ABC, no prior fulvestrant	Alpelisib + fulvestrant (*n = *169) Placebo + fulvestrant (*n = *172)	Primary: PFS in *PIK3CA*m Secondary: OS in *PIK3CA*m	PFS: 0.65 (0.50-0.85; *P <* .001) OS: 0.86 (0.64-1.15; *P =* .15)	
**EPIK-B5** ^54^	1-2L	*PIK3CA*m	*PIK3CA*m, progression on/after CDK4/6i + AI	Alpelisib + fulvestrant (*n = *94) Placebo + fulvestrant (*n = *94)	Primary: BICR-assessed PFS Secondary: OS	PFS: 0.52 (0.37-0.72; *P *< .0001) OS: 0.64 (0.41-0.99; *P *= .021)	
**CAPItello-291** ^55^ ^,^ ^56^	1-4L	All comers (and subgroup with *PIK3CA/AKT1/PTEN* alterations)	Prior AI ± CDK4/6i	Capivasertib + fulvestrant (*n = *355) Placebo + fulvestrant (*n = *353)	Dual primary: PFS in ITT and AKT pathway-altered tumors Secondary: OS	PFS in ITT: 0.60 (0.51-0.71; *P <* .001) PFS in AKT pathway-altered: 0.50 (0.38-0.65; *P <* .001) OS in ITT: 1.00 (0.83-1.19) OS in AKT pathway-altered: 0.83 (0.63-1.10)	PFS benefit independent of alteration type (*AKT1*, *PIK3CA*, or *PTEN*)^57^
**INAVO120** ^58^ ^,^ ^59^	1L	*PIK3CA*m	Relapse during or ≤12 months after adjuvant ET	Inavolisib + palbociclib + fulvestrant (*n = *161) Placebo + palbociclib + fulvestrant (*n = *164)	Primary: PFS Secondary: OS	PFS: 0.43 (0.32-0.59; *P <* .001) OS: 0.67 (0.48-0.94; *P =* .02)	
**VIKTORIA-1** Study 1^60^	≥2L	*PIK3CA*-wildtype	Progression on/after CDK4/6i + AI, ≤2 prior lines of ET, no prior CT for ABC	Gedatolisib + palbociclib + fulvestrant (*n = *131) Gedatolisib + fulvestrant (*n = *130) Fulvestrant (*n = *131)	Primary: BICR-assessed PFS (triplet vs fulvestrant and doublet vs fulvestrant)	PFS: 0.24 (0.17-0.35; *P <* .0001) triplet vs fulvestrant and 0.33 (0.24-0.48; *P <* .0001) doublet vs fulvestrant	
VIKTORIA-1 Study 2^61^	1–3L	*PIK3CA*m	Progression on/after CDK4/6i + AI	Gedatolisib + palbociclib + fulvestrant (*n* = 155) Gedatolisib + fulvestrant (*n* = 52) Alpelisib + fulvestrant (*n* = 155)	Primary: BICR-assessed PFS (triplet vs alpelisib/fulvestrant)	PFS: 0.50 (0.37-0.68; *P* < .0001) triplet vs alpelisib + fulvestrant	

Abbreviations: 1-4L, first–fourth line; ABC, advanced breast cancer; AI, aromatase inhibitor; BICR, blinded independent central review; CDK4/6i, CDK4/6 inhibitor; CI, confidence interval; CT, chemotherapy; ET, endocrine therapy; ITT, intention to treat; OS, overall survival; PFS, progression-free survival.

## Biomarker-driven therapies

While the options above are generally used in broad “all-comer” populations, targeted therapies (PI3K inhibitors, AKT inhibitors, selective estrogen receptor degraders [SERDs], PARP inhibitors, and proteolysis-targeting chimeras [PROTACs]) largely rely on the presence of a specific marker or genetic alteration to predict benefit. Generally, the benefit:risk profile is most favorable in biomarker-selected populations, and in routine practice, biomarker testing usually drives the use of targeted therapies after progression on CDK4/6i therapy.^65^

### PI3K/AKT pathway inhibitors

In patients with *PIK3CA-*mutated tumors (∼40% of HR+ HER2– ABCs^66^), the PI3Kα-specific inhibitor alpelisib significantly improves PFS^52^ (but not OS^53^) when added to fulvestrant after progression on an AI (Table 1). As expected when SOLAR-1 recruited, 94% of patients were CDK4/6i-naïve.^52^ However, the recent EPIK-B5 trial in patients with progression on/after a CDK4/6i and AI showed improved outcomes with the addition of alpelisib to fulvestrant.^54^ Specific alpelisib side effects include severe hypersensitivity, severe cutaneous adverse reactions, hyperglycemia (requiring fasting plasma glucose and HbA1c monitoring and potentially metformin premedication), rash, pneumonitis, and diarrhea/colitis.^67^^,^^68^

AKT pathway activation is implicated in ET resistance. In the phase 3 CAPItello-291 trial, adding the AKT inhibitor capivasertib to fulvestrant significantly improved PFS^55^ with a non-significant numerical improvement in OS^56^ in patients with HR+ HER2– ABC that had progressed during/after AI treatment with/without a CDK4/6i. PFS benefit from capivasertib was seen in both the intention-to-treat (ITT) population and the subgroup (41% of patients) with AKT pathway alterations, independent of the alteration type (*AKT1*, *PIK3CA*, or *PTEN*).^57^ However, the numerical OS effect was seen only in the population with *PIK3CA/AKT1/PTEN*-altered tumors.^56^ Analyses of patient-reported outcomes showed delayed deterioration in global health status/quality of life and little impact on other subscales, except for diarrhea symptoms.^69^ Severe cutaneous reactions, hyperglycemia, and diarrhea require proactive management.^70^ Patients receiving capivasertib require blood glucose monitoring; antidiarrheal medication and increased oral fluids are recommended.

Another therapy targeting the PI3K pathway is inavolisib, which shows enhanced selective inhibition of p110α and degradation of mutated p110α. In the randomized phase 3 INAVO120 trial, both PFS and OS were significantly improved by adding inavolisib to palbociclib and fulvestrant in patients with *PIK3CA*-mutated HR+ HER2– ABC that had recurred during/within 12 months of adjuvant ET.^58^^,^^59^ Median PFS was doubled with the inavolisib triplet versus a palbociclib/fulvestrant doublet.^58^ In clinical practice, however, the triplet’s safety profile (specifically, hyperglycemia, stomatitis, and diarrhea) may limit use to higher-risk patients able to tolerate intensive therapy. For example, INAVO120 excluded patients with type 1 or 2 diabetes requiring ongoing treatment, a fasting glucose level of <126 mg/dL, or a glycated hemoglobin level of <6.0%. Before initiating inavolisib treatment, fasting plasma glucose and HbA1c testing are required, and anti-hyperglycemic medication may be necessary.

The trials above focus on patients with PI3K pathway-altered tumors. The intravenous agent gedatolisib, which inhibits the PI3K/AKT/mTOR pathway, has been evaluated in both *PIK3CA*-wildtype and *PIK3CA*-mutated HR+ HER2– populations. In patients with *PIK3CA*-wildtype HR+ HER2– ABC, gedatolisib, with or without palbociclib, significantly improved PFS when added to fulvestrant as second-line therapy after a CDK4/6i in the VIKTORIA-1 trial (Table 1).^60^ Notable adverse effects include stomatitis, rash, and hyperglycemia.^71^ Practical considerations include the administration schedule (weekly intravenous infusion of gedatolisib, monthly intramuscular injection of fulvestrant, and daily oral palbociclib) and the need for prophylactic steroid mouthwash and antihistamines. Recently reported results from the cohort with *PIK3CA*-mutated tumors demonstrated significant PFS benefit with gedatolisib/fulvestrant, with or without palbociclib, versus an alpelisib/fulvestrant doublet.^61^

### Selective estrogen receptor degraders

For many years the only approved SERD was fulvestrant, which demonstrated superior PFS (but not OS) over anastrozole in ET-naïve postmenopausal women with non-visceral disease.^72^^,^^73^ Fulvestrant has poor bioavailability, limiting overall efficacy; it requires monthly clinic visits for intramuscular injection, and administration is frequently associated with inconvenience and repeated injection-site reactions.^74^ To overcome the burden of intramuscular administration and the modest efficacy of fulvestrant, a new generation of more potent, oral SERDs was developed.^75^

In the open-label phase 3 EMERALD trial in CDK4/6i-pretreated patients, elacestrant monotherapy significantly improved PFS versus standard-of-care ET. Benefit in the ITT population was driven by the more marked effect in the *ESR1*-mutated population (Table 2),^76^ leading to regulatory approval in a biomarker-selected population.^82^ At the final analysis, OS differences between treatment arms were not statistically significant in either the ITT population or the subgroup with *ESR1*-mutated tumors (48% of patients).^82^ Nevertheless, real-world data support a durable clinical benefit from elacestrant, particularly in earlier treatment lines and in patients with extended previous exposure to ET.^83^ The risk of dyslipidemia with elacestrant necessitates lipid profile monitoring before and during treatment.^84^

**Table 2.** Summary of results from key clinical trials evaluating oral SERDs and PROTACs.

Study name	Setting	Biomarker	Eligibility	Treatment	Endpoints	Hazard ratio (95% CI)	Translational findings
**EMERALD** ^76^	2-3L	All comers	Prior CDK4/6i, 1-2 lines of ET, ≤1 line of chemotherapy	Elacestrant (*n = *239) Single-agent ET (*n = *238)	Primary: BICR-assessed PFS in ITT and *ESR1*m	PFS in ITT: 0.70 (0.55-0.88; *P =* .002) PFS in *ESR1*m: 0.55 (0.39-0.77; *P =* .0005)	
**EMBER-3** ^77^ ^,^ ^78^	1/2L	All comers	Progression on ET with/without CDK4/6i (or within 12 months after [neo]adjuvant ET)	Imlunestrant (*n = *331) Imlunestrant + abemaciclib (*n = *213) ET (*n = *330)	Primary: PFS in *ESR1*m and ITT (I vs ET) PFS in ITT (IA vs I) Secondary: OS	PFS I vs ET in *ESR1*m (restricted mean: 7.9 vs 5.4 months; *P <* .001) PFS I vs ET in ITT: 0.87 (0.72-1.04; *P =* .12) PFS IA vs I in ITT: 0.57 (0.44-0.73; *P <* .001) Interim OS I vs ET in *ESR1*m: 0.60 (0.43-0.86, not significant) Interim OS IA vs ET in ITT: 0.82 (0.59-1.16)	Consistent benefit in subgroups with *ESR1* and/or PI3K-pathway mutated tumors^79^
**evERA** ^62^	1-3L	All comers	Progression/relapse on/after CDK4/6i and ET	Giredestrant + everolimus (*n = *183) SOC ET + everolimus (*n = *190)	Co-primary: PFS in ITT and *ESR1m*	PFS in ITT: 0.56 (0.44-0.71; *P <* .0001) PFS in *ESR1*m: 0.38 (0.27-0.54; *P <* .0001)	
**SERENA-6** ^80^	1L	*ESR1*m detected in ctDNA	Molecular but not radiologic progression on CDK4/6i and AI	Camizestrant + continued CDK4/6i (*n = *157) Continued AI + CDK4/6i (*n = *158)	Primary: PFS	0.44 (0.31-0.60; *P <* .00001)	
**VERITAC-2** ^81^	2L	All comers (and subgroup with *ESR1*m)	Prior CDK4/6i and 1-2 prior lines of ET	Vepdegestrant (*n = *313) Fulvestrant (*n = *311)	Primary: BICR-assessed PFS (*ESR1*m and ITT)	PFS in *ESR1*m: 0.58 (0.43-0.78; *P <* .001) PFS in ITT: 0.83 (0.69-1.01); *P =* .07)	

Abbreviations: 1-3L, first–third line; AI, aromatase inhibitor; BICR, blinded independent central review; CDK4/6i, CDK4/6 inhibitor; CI, confidence interval; ctDNA, circulating tumor DNA; ET, endocrine therapy; ITT, intention to treat; OS, overall survival; PFS, progression-free survival; PROTAC, proteolysis-targeting chimera; SERD, selective estrogen receptor degrader; SOC, standard of care.

The three-arm open-label randomized phase 3 EMBER-3 trial evaluated the oral SERD imlunestrant alone or combined with abemaciclib in patients with progression during/after AI therapy with/without a CDK4/6i.^77^ PFS was significantly improved with single-agent imlunestrant versus standard ET (exemestane or fulvestrant) in patients with *ESR1*-mutated tumors, regardless of treatment line (first- or second-line), *PIK3CA* mutation status, and previous CDK4/6i,^85^ but not in the ITT monotherapy population. PFS was also significantly improved with imlunestrant plus abemaciclib versus imlunestrant alone, irrespective of *ESR1*, *PIK3CA* mutation or co-mutation status, or treatment line.^85^ PFS benefit was consistent across clinically relevant subgroups, including prior CDK4/6i exposure (65% of the analysis population), type and duration of prior CDK4/6i therapy, and visceral metastases.^78^ OS results are immature; interim OS results showed clinically meaningful improvements in OS, which did not reach statistical significance.^78^ Patient-reported outcomes indicated maintained global health status/quality of life and function across treatment arms, despite a higher incidence of diarrhea in patients receiving imlunestrant plus abemaciclib.^86^ Imlunestrant use was associated with a low or no incidence of clinically relevant toxicities observed with other members of the oral SERD class, including bradycardia, photopsia, prolonged QT interval, and dyslipidemia.^87^^,^^88^

An investigational oral SERD, giredestrant, demonstrated significantly improved PFS in combination with everolimus after CDK4/6i therapy in the open-label evERA phase 3 trial in the second-line setting.^62^ In both co-primary endpoint populations (ITT and *ESR1*-mutated, representing 55% of patients), PFS was improved with giredestrant plus everolimus versus standard-of-care ET plus everolimus.^62^ At 67% maturity, interim OS results showed a signal in the ITT population. The most common grade ≥3 adverse events with the giredestrant–everolimus combination were consistent with the well-established safety profile of everolimus.^62^ Prophylactic mouthwashes are strongly recommended to minimize everolimus-associated stomatitis.^89^ Recently presented results from the randomized phase 3 persevERA trial showed that giredestrant plus palbociclib did not significantly improve PFS versus letrozole and palbociclib in the first-line setting.^90^

The trials described above explored the introduction of a new therapy on evidence of clinical progression. A novel approach is to switch treatment at the first molecular sign of progression. In the SERENA-6 trial, patients who had received ≥6 months of first-line AI and CDK4/6i therapy were randomized to continue the same therapy on detection of *ESR1* mutations in circulating tumor (ct)DNA (without radiologic progression) or switch to camizestrant with continued CDK4/6i.^80^ PFS was significantly improved in patients switching to camizestrant, and deterioration in patient-reported global health status/quality of life was significantly delayed. These results suggest that both baseline and dynamic *ESR1* mutation status may be important in guiding treatment changes during CDK4/6i therapy and allow interception of tumor resistance before overt disease progression.

While results from the EMERALD, EMBER-3, evERA, and SERENA-6 trials support a role for oral SERDs, important differences between the trials are critical for interpretation. For example, all patients in EMERALD and evERA were CDK4/6i-pretreated and elacestrant and giredestrant were generally administered in the second-line setting or later, whereas in EMBER-3, 41% of patients in the monotherapy arm were CDK4/6i-naïve, no prior chemotherapy was allowed, and patients received imlunestrant in the first- or second-line setting. In SERENA-6, all patients were still receiving a CDK4/6i and ET, and the oral SERD camizestrant replaced ET before evidence of clinical progression. In EMERALD, the benefit from single-agent SERD in the ITT population was driven by the pronounced effect in the *ESR1*-mutated populations. In EMBER-3 and evERA, SERD combination regimens showed a benefit in the ITT population. The unique safety profiles of the various oral SERDs are associated with differing screening and on-treatment monitoring requirements.^91^ Although cross-trial comparisons should be interpreted cautiously, clinically relevant toxicities observed with other oral SERDs (e.g., bradycardia, photopsia, prolonged QTc interval, and dyslipidemia) were not observed with imlunestrant.^87^

### Proteolysis-targeting chimeras

In the VERITAC-2 phase 3 trial in patients with progression on a CDK4/6i and ET, the oral PROTAC estrogen receptor degrader vepdegestrant demonstrated a statistically significant PFS benefit over fulvestrant in patients with *ESR1*-mutated tumors but not in the ITT population.^81^ Vepdegestrant showed QT interval prolongation in 10% of patients (2% grade 3). Consequently, concomitant use of medications that prolong QT should be considered carefully and if such medication is required, patients should be monitored.

### PARP inhibitors

In patients with germline *BRCA1/2*-mutated HER2– ABC, the PARP inhibitors olaparib and talazoparib may be considered standard of care following progression on anthracycline and/or taxane chemotherapy, significantly improving PFS compared with investigator-selected single-agent chemotherapy in the OlympiAD^92^ and EMBRACA^93^ trials, respectively (Table 3). In both trials, approximately half of the patients had HR+ ABC. Neither trial showed a significant OS benefit in the overall population, although in OlympiAD there was an OS signal with olaparib among patients not previously treated with chemotherapy for ABC.^94^^,^^95^ Myelodysplastic syndrome and acute myeloid leukemia are class effects of PARP inhibitors; pneumonitis, venous thromboembolism, and hepatotoxicity are observed with olaparib^96^ and myelosuppression with talazoparib.^97^

**Table 3.** Summary of key clinical trials of PARP inhibitors.

Study name	Setting	Biomarker	Eligibility	Treatment	Endpoints	Hazard ratio (95% CI)
**OlympiAD** ^92^ ^,^ ^94^	1-3L	g*BRCA1/2*m, HER2–	Anthracycline and taxane, ≤2 prior lines of CT for ABC, ET refractory if HR+	Olaparib (*n = *205) Investigator-selected single-agent CT (*n = *97)	Primary: BICR-assessed PFS Secondary: OS	PFS: 0.58 (0.43-0.80; *P <* .001) PFS in HR+ subgroup: 0.82 (0.55-1.26) OS: 0.90 (0.66-1.23; *P =* .51)
**EMBRACA** ^93^ ^,^ ^95^	1-4L	g*BRCA1/2*m, HER2–	Anthracycline and/or taxane, ≤3 prior lines of CT for ABC, unlimited prior ET	Talazoparib (*n = *287) Investigator-selected single-agent CT (*n = *144)	Primary: BICR-assessed PFS Secondary: OS	PFS: 0.54 (0.41-0.71; *P <* .001) PFS in HR+ subgroup: 0.47 (0.32-0.71) OS: 0.85 (0.67-1.07; *P =* .17)

Abbreviations: 1-4L, first–fourth line; ABC, advanced breast cancer; BICR, blinded independent central review; CI, confidence interval; CT, chemotherapy; ET, endocrine therapy; HR+, hormone receptor positive; OS, overall survival; PFS, progression-free survival.

Real-world analyses have suggested worse outcomes with CDK4/6is in patients with *BRCA1/2*-mutated versus *BRCA1/2*-wildtype tumors.^98–101^ However, pooled subgroup analyses of three MONALEESA phase 3 trials yielded contradictory results, with a greater benefit from ribociclib in the subgroup with *BRCA1/2*-mutated tumors.^102^ One of several hypotheses for worse outcomes is the proximity of *BRCA2* and *RB1* in chromosome 13, which often leads to their simultaneous loss.^103^

## Antibody-drug conjugates

ADCs, which enable more targeted chemotherapy delivery to the tumor by linking a cytotoxic payload (often topoisomerase I) to an antibody (typically HER2 or TROP2 in ABC), are transforming breast cancer management.^104^ It remains to be seen whether ADCs will overturn the dogma of treating patients with ET until disease becomes refractory.

The HER2-directed ADC trastuzumab deruxtecan has demonstrated efficacy in phase 3 trials across several ABC settings (Table 4).^105–108^ In the DESTINY-Breast06 trial, trastuzumab deruxtecan demonstrated significantly improved PFS versus investigator-selected chemotherapy in patients with chemotherapy-naïve ABC.^108^ The TROP2-targeting ADCs sacituzumab govitecan and datopotamab deruxtecan demonstrated significantly improved PFS (and OS for sacituzumab govitecan) versus single-agent chemotherapy in patients with heavily pretreated endocrine-resistant HR+ HER2– ABC.^110–114^ OS (dual primary endpoint) was not significantly improved with datopotamab deruxtecan, although imbalanced subsequent exposure to ADCs complicates interpretation.^113^

**Table 4.** Summary of ADC profiles and phase 3 clinical trial results.

Study name	ADC monoclonal antibody	ADC payload	ADC linker	Setting	Biomarker	Eligibility	Treatment	Endpoints	Hazard ratio (95% CI)	Translational findings
**DESTINY-Breast04** ^105^ ^,^ ^106^	Anti-HER2	Topoisomerase I inhibitor	Tetrapeptide-based cleavable linker	2-3L	HER2-low^a^	1-2 prior lines of CT	Trastuzumab deruxtecan (*n = *373) Investigator-selected CT (*n = *184)	Primary: PFS (HR+) Secondary: PFS (ITT), OS (ITT, HR+)	PFS in HR+: 0.51 (0.40-0.64; *P <* .001) PFS in ITT: 0.50 (0.40-0.63; *P <* .001) OS in ITT: 0.69 (0.55-0.86) OS in HR+: 0.69 (0.55-0.87)	Benefit irrespective of intrinsic subtype (luminal A, luminal B, HER2-enriched), *ESR1* or *PIK3CA* mutation status, or CDK4/6i resistance marker status^107^
**DESTINY-Breast06** ^108^	Anti-HER2	Topoisomerase I inhibitor	Tetrapeptide-based cleavable linker		HER2-low or -ultralow		Trastuzumab deruxtecan (*n = *436) Investigator-selected CT (*n = *430)	Primary: BICR-assessed PFS (HER2-low) Secondary: PFS (ITT), OS	PFS in HER2-low: 0.62 (0.52-0.75; *P <* .001) PFS in ITT: 0.64 (0.54-0.76; *P <* .001) OS in HER2-low: 0.83 (0.66-1.05)	Benefit irrespective of PI3K pathway, *ESR1*, and *BRCA* mutational status^109^
**TROPiCS-02** ^110^ ^,^ ^111^	Anti-TROP-2	SN-38 (active metabolite of irinotecan)	Hydrolyzable CL2A linker	2-5L of CT	None	Prior ET, prior CDK4/6i, 2-4 lines of CT, ≥1 taxane	Sacituzumab govitecan (*n = *272) Investigator-selected CT (*n = *271)	Primary: BICR-assessed PFS Secondary: OS	PFS: 0.66 (0.53-0.83; *P =* .0003) OS: 0.79 (0.65-0.96; *P =* .020)	
**TROPION-Breast01** ^112^ ^,^ ^113^	Anti-TROP2 immunoglobulin G1	Topoisomerase I inhibitor	Plasma-stable tumor-selective cleavable linker		None	Progression on ET, 1-2 prior lines of CT	Datopotamab deruxtecan (*n = *365) Investigator-selected CT (*n = *367)	Primary: BICR-assessed PFS and OS	PFS: 0.63 (0.52-0.76; *P <* .0001) OS: 1.01 (0.83-1.22; *P =* .94)	

Abbreviations: 2-5L, second–fifth line; ADC, antibody-drug conjugate; BICR, blinded independent central review; CDK4/6i, CDK4/6 inhibitor; CI, confidence interval; CT, chemotherapy; ET, endocrine therapy; HR+, hormone receptor positive; ITT, intention to treat; OS, overall survival; PFS, progression-free survival.

a Immunohistochemistry 1+ or 2+/in situ hybridization-negative.

Characteristic adverse effects associated with HER2-directed ADCs include neutropenia, left ventricular dysfunction, interstitial lung disease, cardiotoxicity, and hepatotoxicity with trastuzumab deruxtecan^115^^,^^116^ whereas for TROP2-directed therapy, hypersensitivity and infusion-related reactions, nausea, vomiting, and neutropenia are typical with sacituzumab govitecan^117^^,^^118^ and interstitial lung disease/pneumonitis, ocular adverse reactions, stomatitis, and oral mucositis with datopotamab deruxtecan.^119^^,^^120^

Optimal sequencing strategies for ADCs remain unclear, and better understanding of resistance mechanisms is urgently needed.^121–124^ Randomized data on treatment sequencing are lacking, but retrospective data and recent prospective data suggest that efficacy diminishes through repeated lines of ADCs and may be reduced if ADCs conjugated to the same payload (even with different receptor targets) are used sequentially.^125^^,^^126^ Defining the mechanisms of cross-resistance (e.g., between drugs with similar payloads or sharing the same target) will be critical in designing sequencing strategies, which should incorporate underlying biology and multi-omic biomarker strategies.^127^ The emergence of resistance-associated *TOP1* mutations has been reported in patients treated with ADCs bearing TOP1 payloads.^128^

## Impact of endocrine resistance on the efficacy of different treatment regimens

Besides molecular alterations, other clinical and molecular characteristics related to endocrine resistance may influence the efficacy of these agents. A considerable proportion of patients in the EMERALD^76^ and EMBER-3 (monotherapy)^77^ trials experienced disease progression within 2 months of randomization, indicating enrolment of patients with treatment-resistant disease. By 6 months, approximately 60% had experienced progression. However, much lower proportions were seen with combination strategies (imlunestrant plus abemaciclib in EMBER-3,^77^ giredestrant plus everolimus in evERA^62^). In the BOLERO-2 trial, everolimus showed benefit in patients with *ESR1 D538G* mutations, but not in patients with tumors bearing only *Y537S* mutations or both *D538G* and *Y537S* mutations.^129^ In contrast, elacestrant showed consistent efficacy across these alleles in a large real-world analysis.^130^

In the EMERALD trial, PFS benefit in patients with *ESR1*-mutated tumors appeared to increase with increasing duration of prior CDK4/6i exposure, even in patients with concurrent *PIK3CA* or *TP53* mutations, low HER2 expression, or liver/lung metastases.^131^ Elacestrant use may be particularly appropriate for patients with *ESR1*-mutated tumors and prolonged sensitivity to CDK4/6i, acting as a surrogate for endocrine dependence and sensitivity. In EMBER-3, PFS benefit from combining abemaciclib with imlunestrant was observed regardless of *ESR1*, *PIK3CA*, or co-mutation status and irrespective of line of therapy or the type or duration of prior CDK4/6i; similarly, imlunestrant monotherapy in the *ESR1*-mutant population demonstrated PFS benefit regardless of line of therapy, *PIK3CA* mutation status, and previous CDK4/6i.^77^^,^^78^

## Timing and relevance of molecular testing

As treatment efficacy is influenced by molecular alterations (which may also occur in early-stage disease^132^) and clinicopathological characteristics, molecular testing has become pivotal in selecting treatments with the greatest likelihood of success, and guidelines now recommend tumor next-generation sequencing in HR+ HER2– ABC.^133^ High-throughput sequencing enables the rapid, affordable, and accurate characterization of cancer cell alterations.^38^ Additionally, ctDNA technologies allow the detection of cancer molecular alterations through biofluids, particularly blood, facilitating molecular profiling.^134^^,^^135^ In some cases, current technologies do not provide the same level of detail as tissue analyses,^136^ but nuances between liquid and tissue analyses should be considered carefully, particularly for guideline-based *ESR1* variants, which are detected more accurately by ctDNA analysis.^137^ Consequently, the timing and method of molecular characterization have become critical components of therapeutic strategies for HR+ HER2– ABC.

In first-line therapy, the INAVO120 trial results^58^^,^^59^ support assessing *PIK3CA* mutation status in patients with metastatic progression during/within 12 months of completing adjuvant ET. Results from the SERENA-6 trial^80^ support regular *ESR1* testing during first-line CDK4/6i and ET. For patients who experience metastatic progression during CDK4/6i in the adjuvant or first-line ABC settings, molecular testing may help detect *ESR1*, *PIK3CA*, or *AKT1* mutations or PTEN loss, all of which could be targeted with available therapies. Importantly, conducting molecular characterization on a new sample at relapse facilitates informed treatment decisions. The decision-making process should aim to balance the expected benefits against the risks and side effects of treatment to determine whether a molecularly driven approach (e.g., PI3K/AKT inhibition) or a non-biomarker-guided therapy (e.g., continued ET and CDK4/6i and/or mTOR inhibition) is more appropriate. Given the increasing recognition of disease complexity, treatment decisions based on a single biomarker are likely to be replaced by combinations of clinical and genomic factors used in conjunction in multimodal machine-learning models.^138^

## Suggested clinical management strategies

The clinical trial data above illustrate the importance of assessing ET sensitivity and/or resistance according to biomarkers as well as previous clinical responses. As the treatment landscape becomes increasingly complex and available options expand, thorough workups at initial diagnosis and upon progression of ABC become critical. By identifying markers of resistance and sensitivity, a biomarker-guided approach to treatment selection can be adopted, with the aim of optimizing clinical outcomes with available and emerging therapies. The ultimate goal is to treat patients with the most effective and best-tolerated drug first, giving patients the best quality and duration of life. This raises the question of how best to sequence regimens, which is particularly relevant in situations where a patient may receive many years of clinical care and multiple treatment strategies. Insights on CDK4/6i sequencing from the SONIA trial suggest that in some postmenopausal women with ET-sensitive ABC, single-agent ET followed by a CDK4/6i and ET combination at progression may be reasonable.^139^^,^^140^ Beyond these findings, however, optimal sequencing has not been studied adequately in recent trials and clinical trial data on postprogression therapies are difficult to decipher. Sequencing will become more complex now that CDK4/6is are an established adjuvant therapy, and with the advent of oral SERDs in the early-stage setting.^141^

The prevalence of co-existing *ESR1* and PI3K pathway mutations increases through treatment lines^37^ and is observed in 15%-20% of patients after progression on ET and a CDK4/6i^29^^,^^130^^,^^131^ but, currently, the optimal therapeutic approach in this scenario is unknown. If both *PIK3CA* and *ESR1* mutations are detected at progression, therapies targeting *PIK3CA* mutations could potentially be preferred because of the more dominant signaling activation function of *PIK3CA* over *ESR1* mutations. On the other hand, in patients with *ESR1* and *PIK3CA* co-mutations, subgroup analyses of the EMERALD^131^ and EMBER-3^77^ phase 3 trials support the efficacy of elacestrant and imlunestrant (alone or in combination with abemaciclib), respectively.

Besides carefully evaluating the available efficacy data for different therapeutic options and considering the molecular profile of the disease and its degree of endocrine sensitivity, treatment selection may also be influenced by patient-related factors that are unrelated to breast cancer. For example, trials of PI3K-pathway inhibitors have excluded patients with diabetes, and hyperglycemia requires close monitoring.^142^ Consequently, these agents may not be an option for some individuals, even if their disease harbors a *PIK3CA* mutation. Similarly, frailer patients or those with significant comorbidities may not tolerate combination or chemotherapy-based regimens. Within the oral SERD class, different agents have distinct safety profiles and unique toxicities, such as low-grade bradycardia,^62^^,^^143^ QT interval prolongation,^81^ visual disturbances,^119^ and dyslipidemia,^84^ which may have a significant impact on patients’ quality of life and require careful consideration during treatment decision-making. These events appear with varying incidence between the various oral SERDs (e.g., no photopsia with imlunestrant or giredestrant). Clinicians are urged to refer to practical guidelines for the management of class-specific common side effects (e.g., hyperglycemia, rash, stomatitis, and diarrhea) with PI3K/AKT/mTOR pathway inhibitors.^144^

**Figure 2.**
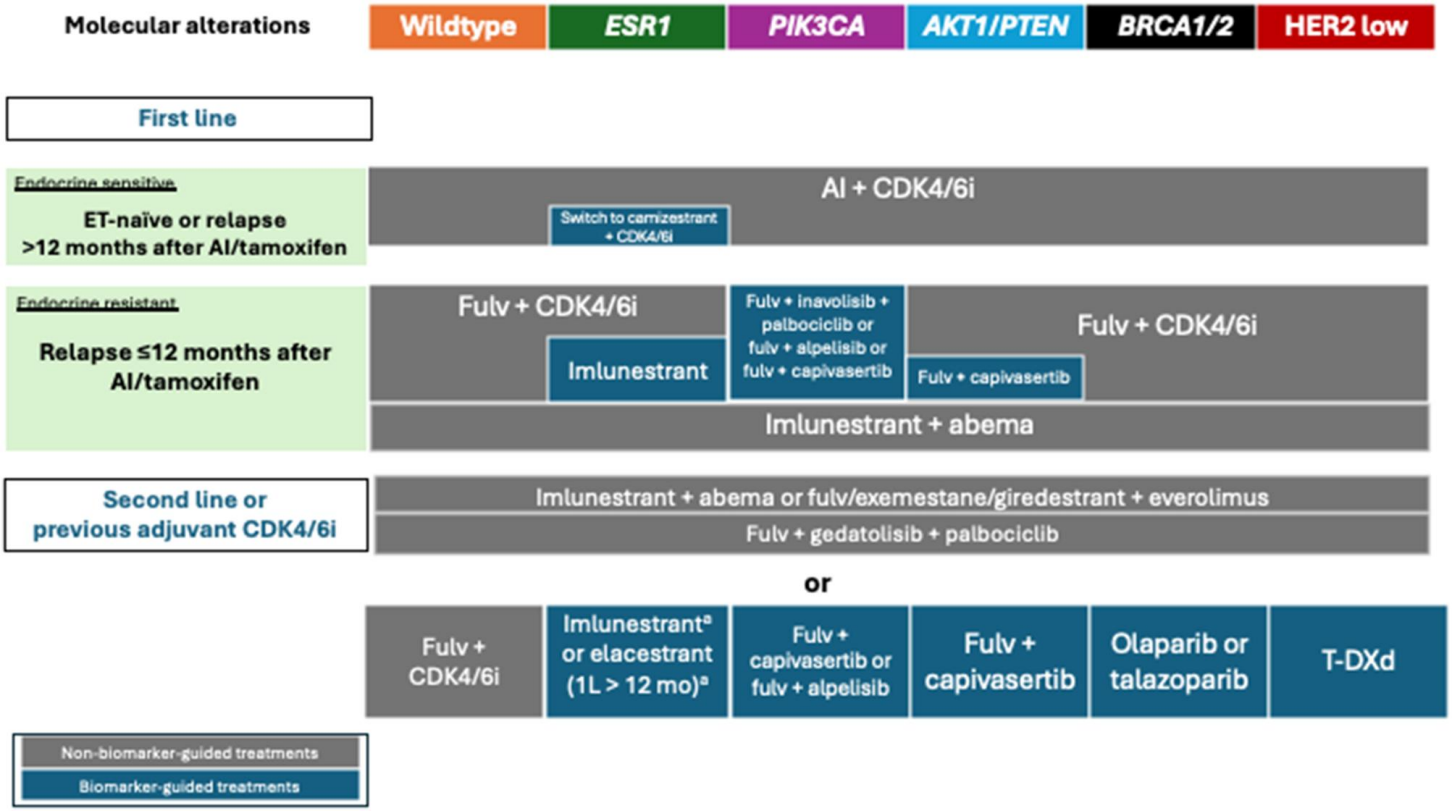
Biomarker-driven treatment selection after progression on a CDK4/6i plus ET. This proposed algorithm should be adapted based on local approvals and reimbursement policies. Preference should be given to clinical trial inclusion where available. ^a^Including *ESR1*/*PIK3CA* co-mutations. Chemotherapy is not mentioned but should be considered for patients in visceral crisis or with no further endocrine options. Abbreviations: 1L, first line; abema, abemaciclib; AI, aromatase inhibitor; CDK4/6i, CDK4/6 inhibitor; ET, endocrine therapy; fulv, fulvestrant; T-DXd, trastuzumab deruxtecan.

In this increasingly segmented space, multiple clinical and biological factors will be considered when selecting the optimal therapy for a patient, including patient preferences. Figure 2 aims to summarize factors that influence treatment selection after progression on a CDK4/6i and ET.

## Unmet needs and future directions

Table 5 summarizes key ongoing trials in ABC expected to report soon and contribute to future evidence-based sequencing strategies.^145^^,^^146^ Looking ahead, pan-mutant isoform-selective PI3Kα inhibitors may offer more tolerable strategies, aiming to avoid some of the off-target toxicities. Emerging therapeutic strategies to overcome CDK4/6i resistance include next-generation approaches such as inhibitors of CDK2, CDK4, CDK7, and KAT6.^147^ The recognition of different populations of patients within HR+ HER2– ABC is revolutionizing our therapeutic approach. Two main concepts are evolving: first, the clear need to develop better molecular biomarkers or combinations of clinical and pathological biomarkers that allow specific patient populations to be selected for innovative strategies (e.g., initiating therapy on emergence of tumor mutations^80^) and to define the optimal timing for introducing new targeted therapies;^138^ and second, the consistent improvement in outcomes observed with combinations versus single-agent ET. Future trial designs should address these two main concepts and aim to understand how treatments should be sequenced to optimize long-term outcomes. This is particularly important considering recent data on switching strategies, where comparison of treatment lines becomes blurred. Artificial intelligence may play a role in optimizing sequential therapy. Developing combinations at an earlier stage of the drug development process will require collaborative efforts involving industry, regulatory agencies, and academia. New combinations may bring new toxicity concerns that must be considered and overcome. Furthermore, a better understanding of complex resistance mechanisms and strategies to reverse or prevent resistance is essential if we are to identify next-generation therapeutic options.^148^^,^^149^ Innovative therapies should also be explored in early-stage disease, where they offer the possibility of cure. Recently the lidERA randomized phase 3 trial demonstrated significantly improved outcomes with adjuvant giredestrant versus standard adjuvant ET.^141^ Other trials of oral SERDs in the adjuvant setting are expected to report in the near future.^150–152^ Such advances require constant reevaluation of treatment algorithms as the treatment history of patients presenting in clinical practice shifts. Finally, the consistent improvement in outcomes (PFS and OS) with the new ADCs raises further questions about sequencing, and ongoing trials of ADCs with different targets and/or payloads may potentially increase complexity. Treatment sequencing is becoming increasingly multifaceted with the wealth of new therapies in HR+ HER2– ABC. Treatment selection should be tailored according to clinical and molecular characteristics while considering patient-focused factors (e.g., administration burden, side-effect profiles).

**Table 5.** Summary of potentially practice-changing key phase 3 trials on the horizon.

Study name	Clinicaltrials.gov identifier	Experimental	Control	Setting	Efficacy endpoints
**pionERA**	NCT06065748	Giredestrant + CDK4/6i	Fulvestrant + CDK4/6i	ER+ HER– ABC progressing on/<12 months after adjuvant ET (1L)	Primary: PFS (*ESR1-*mutant and ITT populations) Secondary: PFS (*ESR1*-wildtype), OS, ORR, DoR, CBR, TTC
**SERENA-4** ^145^	NCT04711252	Camizestrant + palbociclib	Anastrozole + palbociclib	ER+ HER2– ABC (1L)	Primary: PFS Secondary: OS, PFS2, ORR, DoR, TTC, TFST, CBR at 24 weeks, TSST
**VIKTORIA-2**	NCT06757634	Gedatolisib + fulvestrant + CDK4/6i (ribociclib/palbociclib)	Fulvestrant + CDK4/6i (ribociclib/palbociclib)	*PIK3CA*-mutated or -unknown HR+ HER2– ABC progressing <12 months after adjuvant ET (1L)	Primary: PFS Secondary: OS, ORR, DoR, TTR, CBR
**INAVO121**	NCT05646862	Inavolisib + fulvestrant	Alpelisib + fulvestrant	*PIK3CA*-mutated HR+ HER2– ABC with progression during/after CDK4/6i + ET	Primary: BICR-assessed PFS Secondary: OS, BICR-assessed ORR, BOR, DoR, CBR
**PIKALO-2**	NCT07174336	Tersolisib (LY4064809) + CDK4/6i + ET	Placebo + CDK4/6i + ET	*PIK3CA*-mutated HR+ HER2– ABC (1L)	Primary: PFS Secondary: DCR, TTR, DoR, OS, CBR, PFS2, ORR, TTC, CT-free survival
**CULMINATE-2^146^**	NCT05365178	Culmerciclib (TQB3616; CDK2/4/6i) + fulvestrant	Fulvestrant	HR+ HER2– ABC (1L)	Primary: PFS Secondary: BICR-assessed PFS, OS, ORR, CBR, DoR
**FourLight-3**	NCT06760637	Atirmociclib (CDK4/6i) + letrozole	CDK4/6i + letrozole	HR+ HER2– ABC (1L)	Primary: BICR-assessed PFS Secondary: OS, investigator-assessed PFS, DoR
**CAPItello-292**	NCT04862663	Capivasertib + CDK4/6i + fulvestrant	CDK4/6i + fulvestrant	HR+ HER2– ABC	Primary: PFS Secondary: OS, ORR, PFS2, DoR, CBR
**ReDiscover-2**	NCT06982521	Zovegalisib (RLY-2608) + fulvestrant	Capivasertib + fulvestrant	*PIK3CA*-mutated HR+ HER2– ABC after recurrence/progression on/after CDK4/6i	Primary: BICR-assessed PFS (ITT and kinase populations) Secondary: OS, investigator-assessed PFS, ORR, DoR, CBR (all in ITT and kinase populations)
**C4551002**	NCT07062965	PF-07248144 (KAT6 inhibitor) + fulvestrant	Everolimus + ET	HR+ HER2– ABC progressing after CDK4/6i	Primary: BICR-assessed PFS Secondary: OS, BICR-assessed ORR, DoR, CBR
**LAE205INT3101**	NCT04851613	Afuresertib + fulvestrant	Fulvestrant	HR+ HER2– ABC progressing on ET and/or CDK4/6i (or CT for ABC)	Primary: PFS Secondary: BICR-assessed PFS

Abbreviations: 1L, first line; ABC, advanced breast cancer; BICR, blinded independent central review; BOR, best overall response; CBR, clinical benefit rate; CDK4/6i, CDK4/6 inhibitor; CT, chemotherapy; DCR, disease control rate; DoR, duration of response; ER+, estrogen receptor positive; ET, endocrine therapy; HR+, hormone receptor positive; ITT, intention to treat; ORR, objective response rate; OS, overall survival; PFS, progression-free survival; TFST, time to first subsequent therapy; TSST, time to second subsequent therapy; TTC, time to chemotherapy; TTR, time to response.

## Supplementary Material

oyag215_Supplementary_Data

## Data Availability

No new data were generated or analyzed in support of this research.
