# Supplementary material for: Navigating first- and second-line treatment options in hormone receptor-positive HER2-negative advanced breast cancer
Source: Oncologist. 2026 Jun 9;31(8):oyag215. doi: 10.1093/oncolo/oyag215 (PMC13379707; doi:10.1093/oncolo/oyag215)
Supplement: oyag215_Supplementary_Data [file oyag215_supplementary_data.docx]

**Supplemental Table 1.** Summary of key clinical trials of CDK4/6i.

| **Study name** | **Setting** | **Bio-marker** | **Eligibility** | **Treatment** | **Endpoints** | **Hazard ratio (95% CI)** | **Translational findings** |
| --- | --- | --- | --- | --- | --- | --- | --- |
| MONARCH 2^1, 2^ | 1–2L | All comers | ET refractory, ≤1 prior line of ET, no CT | Abemaciclib + fulvestrant (*n =* 446)  Placebo + fulvestrant (*n =* 223) | Primary: PFS  Secondary: OS | PFS: 0.55 (0.45–0.68; *P =* .001)  OS: 0.76 (0.61–0.95; *P =* .01) | Benefit from CDK4/6i irrespective of *ESR1* or *PIK3CA* mutation status^3^ |
| PALOMA-2^4, 5^ | 1L | All comers | Postmenopausal, no prior therapy for ABC | Palbociclib + letrozole (*n =* 444)  Placebo + letrozole (*n =* 222) | Primary: PFS  Secondary: OS | PFS: 0.58 (0.46–0.72; *P <* .001)  OS: 0.96 (0.78–1.18; *P =* .34) |  |
| PALOMA-3^6, 7^ | 1–2L | All comers | ET refractory | Palbociclib + fulvestrant (*n =* 347)  Placebo + fulvestrant (*n =* 174) | Primary: PFS  Secondary: OS | PFS: 0.42 (0.32–0.56; *P <* .001)  OS: 0.81 (0.65–0.99; exploratory) | Benefit from CDK4/6i irrespective of *ESR1* or *PIK3CA* mutation status^7^ ^8^ |
| MONALEESA-7^9, 10^ | 1–2L | All comers | Pre- or perimenopausal, ≤1 line of CT for ABC | Ribociclib + ET (*n =* 335)  Placebo + ET (*n =* 337) | Primary: PFS  Secondary: OS | PFS: 0.55 (0.44–0.69; *P <* .0001)  OS: 0.71 (0.54–0.95; *P <* .01) |  |
| MONALEESA-2^11, 12^ | 1L | All comers | No prior systemic therapy for ABC, DFI ≥12 months | Ribociclib + letrozole (*n =* 334)  Placebo + letrozole (*n =* 334) | Primary: PFS  Secondary: OS | PFS: 0.56 (0.43–0.72; *P* = 3.29×10^−6^)  OS: 0.76 (0.63–0.93; *P =* .008) | Benefit from CDK4/6i irrespective of *PIK3CA* mutation status^13^ |
| MONALEESA-3^14, 15^ | 1–2L | All comers | Postmenopausal, treatment-naïve or 1 prior line of ET | Ribociclib + fulvestrant (*n =* 484)  Placebo + fulvestrant (*n =* 242) | Primary: investigator-assessed PFS  Secondary: OS | PFS: 0.59 (0.48–0.73; *P <* .001)  OS: 0.67 (0.50–0.90) |  |
| MONARCH 3^16-18^ | 1L | All comers | Postmenopausal, no prior systemic therapy for ABC | Abemaciclib + NSAI (*n =* 328)  Placebo + NSAI (*n =* 165) | Primary: PFS  Secondary: OS | PFS: 0.54 (0.42–0.70; *P <* .0001)  OS: 0.80 (0.64–1.02; *P =* .066) | Benefit from CDK4/6i irrespective of *ESR1* or *PIK3CA* mutation status; acquired *ESR1* mutations less frequent in patients progressing on abemaciclib than on placebo.^19^ |

1–2L = first–second line; ABC = advanced breast cancer; AI = aromatase inhibitor; CI = confidence interval; CT = chemotherapy; DFI = disease-free interval; ET = endocrine therapy; NSAI = non-steroidal aromatase inhibitor; OS = overall survival; PFS = progression-free survival.

**References**

1 Sledge GW, Jr., Toi M, Neven P et al. MONARCH 2: Abemaciclib in combination with fulvestrant in women with HR+/HER2- advanced breast cancer who had progressed while receiving endocrine therapy. J Clin Oncol 2017; 35 (25): 2875-2884.

2 Sledge GW, Jr., Toi M, Neven P et al. The effect of abemaciclib plus fulvestrant on overall survival in hormone receptor-positive, ERBB2-negative breast cancer that progressed on endocrine therapy - MONARCH 2: a randomized clinical trial. JAMA Oncol 2020; 6 (1): 116-124.

3 Tolaney SM, Toi M, Neven P et al. Clinical significance of *PIK3CA* and *ESR1* mutations in circulating tumor DNA: analysis from the MONARCH 2 study of abemaciclib plus fulvestrant. Clin Cancer Res 2022; 28 (8): 1500-1506.

4 Finn RS, Martin M, Rugo HS et al. Palbociclib and letrozole in advanced breast cancer. N Engl J Med 2016; 375 (20): 1925-1936.

5 Slamon DJ, Diéras V, Rugo HS et al. Overall survival with palbociclib plus letrozole in advanced breast cancer. J Clin Oncol 2024; 42 (9): 994-1000.

6 Turner NC, Ro J, André F et al. Palbociclib in hormone-receptor-positive advanced breast cancer. N Engl J Med 2015; 373 (3): 209-219.

7 Cristofanilli M, Rugo HS, Im SA et al. Overall survival with palbociclib and fulvestrant in women with HR+/HER2- ABC: updated exploratory analyses of PALOMA-3, a double-blind, phase 3 randomized study. Clin Cancer Res 2022; 28: 3433-3442.

8 Fribbens C, O'Leary B, Kilburn L et al. Plasma *ESR1* mutations and the treatment of estrogen receptor-positive advanced breast cancer. J Clin Oncol 2016; 34 (25): 2961-2968.

9 Tripathy D, Im SA, Colleoni M et al. Ribociclib plus endocrine therapy for premenopausal women with hormone-receptor-positive, advanced breast cancer (MONALEESA-7): a randomised phase 3 trial. Lancet Oncol 2018; 19 (7): 904-915.

10 Im SA, Lu YS, Bardia A et al. Overall survival with ribociclib plus endocrine therapy in breast cancer. N Engl J Med 2019; 381 (4): 307-316.

11 Hortobagyi GN, Stemmer SM, Burris HA et al. Ribociclib as first-line therapy for HR-positive, advanced breast cancer. N Engl J Med 2016; 375 (18): 1738-1748.

12 Hortobagyi GN, Stemmer SM, Burris HA et al. Overall survival with ribociclib plus letrozole in advanced breast cancer. N Engl J Med 2022; 386 (10): 942-950.

13 Hortobagyi GN, Stemmer SM, Burris HA et al. Updated results from MONALEESA-2, a phase III trial of first-line ribociclib plus letrozole versus placebo plus letrozole in hormone receptor-positive, HER2-negative advanced breast cancer. Ann Oncol 2018; 29 (7): 1541-1547.

14 Slamon DJ, Neven P, Chia S et al. Phase III randomized study of ribociclib and fulvestrant in hormone receptor-positive, human epidermal growth factor receptor 2-negative advanced breast cancer: MONALEESA-3. J Clin Oncol 2018; 36 (24): 2465-2472.

15 Neven P, Fasching PA, Chia S et al. Updated overall survival from the MONALEESA-3 trial in postmenopausal women with HR+/HER2- advanced breast cancer receiving first-line ribociclib plus fulvestrant. Breast Cancer Res 2023; 25 (1): 103.

16 Goetz MP, Toi M, Huober J et al. Abemaciclib plus a nonsteroidal aromatase inhibitor as initial therapy for HR+, HER2– advanced breast cancer: final overall survival results of MONARCH 3. Ann Oncol 2024; 35 (8): 718-727.

17 Johnston S, Martin M, Di Leo A et al. MONARCH 3 final PFS: a randomized study of abemaciclib as initial therapy for advanced breast cancer. NPJ Breast Cancer 2019; 5: 5.

18 Goetz MP, Toi M, Campone M et al. MONARCH 3: abemaciclib as initial therapy for advanced breast cancer. J Clin Oncol 2017; 35 (32): 3638-3646.

19 Goetz MP, Hamilton EP, Campone M et al. Landscape of baseline and acquired genomic alterations in circulating tumor DNA with abemaciclib alone or with endocrine therapy in advanced breast cancer. Clin Cancer Res 2024; 30 (10): 2233-2244.
